# Building Health Sciences Library Collections: A Handbook

**DOI:** 10.5195/jmla.2024.1927

**Published:** 2024-07-01

**Authors:** Barnaby Nicolas

**Affiliations:** 1 barnaby.nicolas@nyulangone.org, Hollis Health Sciences Library, NYU Grossman Long Island School of Medicine, Mineola, NY

Referring to developing and managing health sciences library collections as an art would not be a hyperbolic statement. It is a detailed process and ongoing cycle in which librarians acquire skills and refine these skills through practice. Building Health Sciences Library Collections: A Handbook provides guidance to librarians, both new and experienced, working within a collections unit or as a solo librarian, on how to effectively build and maintain library collections. In addition to informing readers about the value of developing a strategy and employing a methodology, the editors and chapters' authors advise that collection development requires time, dedication, and collaboration with technical services employees, liaisons, administration, departments, and other relevant groups within an organization. At the end of each chapter, a list of suggested resources to acquire and a list of suggested resources to consult for further reading are provided. The type of resources within these sections can include books, journals, electronic information resources, bibliographies, or all four.

The first chapter of Building Health Sciences Library Collections: A Handbook is penned by Megan Inman who is one of the editors and does a great job of presenting an overview about collection development. Inman cites recent studies and surveys to provide evidence of the selection trends within health sciences librarianship, e.g., electronic access being the overwhelming preferred format. These trends also influence libraries to reassess their purchasing model such as electing to pursue a subscription-based model rather than a perpetual access model since it would supply them with the latest editions to existing titles within the collection and new resources altogether.

Subsequent chapters focus on developing collections within the health sciences disciplines of medicine, nursing, and allied health, respectively. These chapters serve as practical guides for librarians as they can explore reputable resources or materials worth adding to their collections to support the needs of their users. Chapter 5 offers bibliographies for various specialties within the medicine discipline, clinical reasoning, doctoring, and evidence-based medicine. The materials include books, databases, and differential and point-of-care tools. This chapter also provides a bibliography to support different medical roles: students, residents, fellows, and clinicians. Very similar to Chapter 5, Chapter 6 highlights specific resources in various types and formats that serve the needs of nurses. This structure repeats in Chapter 7 as it provides an overview of online free resources of various formats for allied health professionals and offers bibliographies for eleven allied health professional roles.

In addition to offering guidance on how to develop subject specific collections, the book dedicates its Chapter 2 to developing diverse and inclusive collections with sound strategies and discusses the positive impact this effort can have on the patient experience and patients' trust in the medical profession. An inclusive collection that promotes DEI and the scholarship of researchers from diverse backgrounds or viewpoints, the book asserts, provides medical professionals an opportunity to learn more about the experiences of underrepresented groups. Furthermore, the chapter also endorses building a collection of diverse materials to support protected groups such as licensing adaptive technologies for people living with visual impairment. The chapter also acknowledges the challenges libraries may face when attempting to grow diverse and inclusive collections due to lack of organizational buy-in and the amount of time and dedication that is required of a librarian to research and identify relevant resources.

A critical process in managing library collections is assessing the inventory and deaccessioning. Chapter 3 focuses on deaccessioning books in the health sciences library environment and finds it to be imperative in order to ensure currency in content, upkeep of the condition of physical books in the stacks, alignment with the academic curriculum, and fitting within the library space planning. The chapter advises libraries to use their collection development policies as roadmaps for the deselection process and inventory reports to capture the overall status of the collections to determine what needs to be weeded from their collections. The book also offers suggestions on how to repurpose the library space after deaccessioning and what to do with the de-selected books, such as, launching a library book sale or donating to socially responsible and second-hand bookstores.

Building Health Sciences Library Collections: A Handbook is a very comprehensive handbook that covers the collection development process. Seasoned librarians can expect to find commonalities between what the editors and authors advise with their own library practices and serve as validation of their processes. They may also find there to be valuable nuggets of new information in reading Chapter 4 to be mindful about emerging topics when building a collection such as health humanities, graphic medicine, children's books, and cookbooks; and to also consider supporting Open Educational Resources. New librarians will have a great appreciation for the perspectives and expertise offered by the editors and authors. They may also find it useful to consult one of the materials referenced in chapter 3, “Managing a Collection Budget,” of Health Sciences Collection Management for the Twenty-First Century by Susan K. Kendall (Rowman & Littlefield Publishers, 2018) to learn more about negotiating strategies with publishers and vendors as a supplement to reading this book.

